# The Super Thin External Pudendal Artery Free Flap for Buccal Reconstruction: A Case Report

**DOI:** 10.1002/micr.70261

**Published:** 2026-07-24

**Authors:** Olivier Mathieu, Leo Ouhayoun, Nicolas Walhin, Jean‐François Honart, Stéphane Temam, Nicolas Leymarie

**Affiliations:** ^1^ Department of Plastic Surgery Gustave Roussy Cancer Campus Villejuif France; ^2^ Department of Otorhinolaryngology and Head and Neck Surgery Gustave Roussy Cancer Campus Villejuif France

## Abstract

Reconstruction of buccal mucosal defects requires thin, pliable, and well‐vascularized tissue to preserve oral function and cheek mobility. While the radial forearm free flap remains widely used, alternative ultra‐thin options are needed, particularly in obese patients where conventional perforator flaps may be excessively thick. We report the case of a 59‐year‐old obese man (BMI 32 kg/m^2^) undergoing resection of a superficial squamous cell carcinoma of the left buccal mucosa, resulting in a wide 6 × 6 cm defect. Reconstruction was performed using a super‐thin external pudendal artery (STEPA) free flap harvested from the hemiscrotum. The fasciocutaneous flap, measuring 6 × 6 cm, demonstrated uniform near‐millimetric thickness (2–3 mm), allowing direct intraoral inset without the need for thinning. Microvascular anastomoses were performed to the facial vessels, and donor‐site closure was achieved primarily. The postoperative course was uneventful, with early enteral feeding, progressive oral intake from day 10, and no complications. At 3 months of follow‐up, the flap showed complete integration with preserved cheek mobility and satisfactory mouth opening. This case highlights that the STEPA free flap may represent a useful ultra‐thin reconstructive option for buccal mucosal defects in selected patients, particularly when minimal flap thickness is required.

## 1 Introduction

Reconstruction of buccal mucosal defects requires the use of thin, pliable, and well‐vascularized tissue capable of restoring lining while preserving cheek mobility, oral continence, and mastication. The radial forearm free flap (RFFF) remains widely used for these indications (Soutar and McGregor 1986), but its donor‐site morbidity—including tendon exposure, need for skin graft, functional limitation, and visible scarring (Orlik et al. 2014)—makes alternative thin flaps increasingly attractive. Perforator‐based flaps such as the anterolateral thigh, thoracodorsal artery perforator, or superficial circumflex iliac artery perforator flaps may be raised as “thin” or “ultra‐thin” variants; however, these techniques are technically demanding and often remain excessively thick for buccal mucosal reconstruction, particularly in obese patients. Recent anatomical work has characterized the territory of the external pudendal artery, demonstrating a reliable vascular pedicle supplying ultra‐thin scrotal and inguinal skin with highly favorable mechanical properties (Phoon et al. 2014). The so‐called super‐thin external pudendal artery (STEPA) flap offers laminar tissue closely comparable to the RFFF but with minimal donor‐site sequelae, a hidden scar, and surprisingly consistent thickness even in overweight patients (Phoon et al. 2014; Kiranantawat et al. 2018; Dupret‐Bories et al. 2019). Additional anatomical work has further supported the feasibility of EPA‐based free flaps for buccopharyngeal reconstruction, including a cadaveric study in women demonstrating a consistent pedicle, an extensive angiosome, and favorable flap dimensions for intraoral use (Benbassat et al. 2022).

Although this territory is already employed in pedicled configurations for perineal and genital reconstruction (Del Corral et al. 2024), its use as a microvascular free flap remains exceptionally uncommon. Only a handful of reports describe their use in extremity reconstruction (Kiranantawat et al. 2018), and a single publication has addressed their application in head and neck oncologic surgery (Dupret‐Bories et al. 2019), where their thinness and elasticity may represent a major reconstructive advantage.

We present a case of extended buccal mucosa defect reconstruction using a scrotal fasciocutaneous free flap based on the external pudendal artery. This report emphasizes the technical features of the flap, its advantages in intraoral reconstruction, and its potential position among thin flaps in head and neck surgery.

Although this territory is already employed in pedicled configurations for perineal and genital reconstruction (Del Corral et al. 2024), its use as a microvascular free flap remains extremely limited, with only isolated case reports available in the literature (Kiranantawat et al. 2018) and no published clinical series to date. In head and neck reconstruction, its application has been reported only once for oropharyngeal reconstruction (Dupret‐Bories et al. 2019).

We present a case of extended buccal mucosa defect reconstruction using a STEPA free flap. To our knowledge, this is the first report describing its use for buccal mucosal reconstruction, and we aim to highlight its potential interest in clinical situations requiring ultra‐thin and pliable intraoral lining.

## 2 Case Report

A 59‐year‐old man with obesity (BMI 32 kg/m^2^) was referred for management of a superficial squamous cell carcinoma of the left buccal mucosa. Imaging demonstrated a lesion confined to the mucosa and superficial fibers of the buccinator muscle, without mandibular involvement or cervical lymph node metastasis. Surgical management combining transoral wide excision, ipsilateral selective neck dissection, and free‐flap reconstruction was recommended.

Given the patient's obesity, conventional perforator‐based free flaps commonly used for intraoral reconstruction—such as the anterolateral thigh, thoracodorsal artery perforator, profunda artery perforator, or superficial circumflex iliac artery perforator flaps—were considered suboptimal due to their excessive thickness for buccal mucosal lining, even when raised as “thin” or “ultra‐thin” variants. Extensive thinning would have been required, with an increased risk of fat necrosis and compromised perfusion. A RFFF was not favored because of its well‐documented donor‐site morbidity and the absence of clear functional benefit in this context. For these reasons, an ultra‐thin fasciocutaneous flap based on the external pudendal artery was selected to provide a naturally thin, pliable lining while minimizing donor‐site sequelae.

Following selective neck dissection with preparation of recipient vessels, transoral excision encompassed the entire involved buccal mucosa. Although tumor infiltration was limited, the superficial extension was broad, resulting in a large mucosal defect requiring an ultra‐thin and flexible lining to preserve cheek mobility and oral function.

**FIGURE 1:**
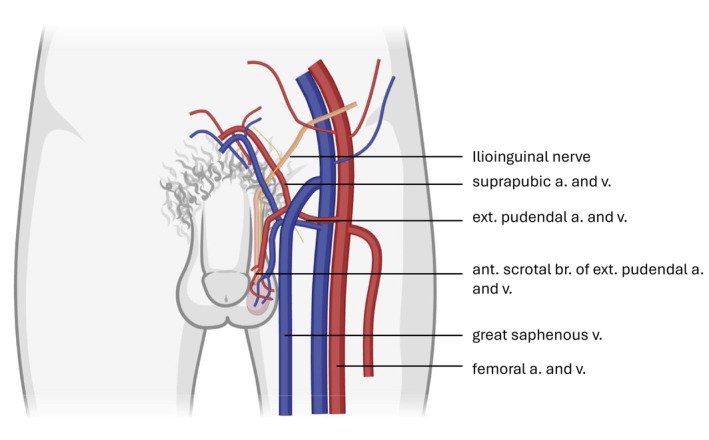
Vascular anatomy of the super‐thin external pudendal artery (STEPA) flap. Schematic illustration showing the external pudendal artery and vein, their suprapubic and anterior scrotal branches, and their relationship to the femoral artery and vein, great saphenous vein, and ilioinguinal nerve. The scrotal skin paddle, supplied by the external pudendal vessels, shows a uniform near‐millimetric thickness (approximately 2–3 mm), offering exceptional pliability and ideal characteristics for intraoral lining without the need for primary thinning.

**FIGURE 2:**
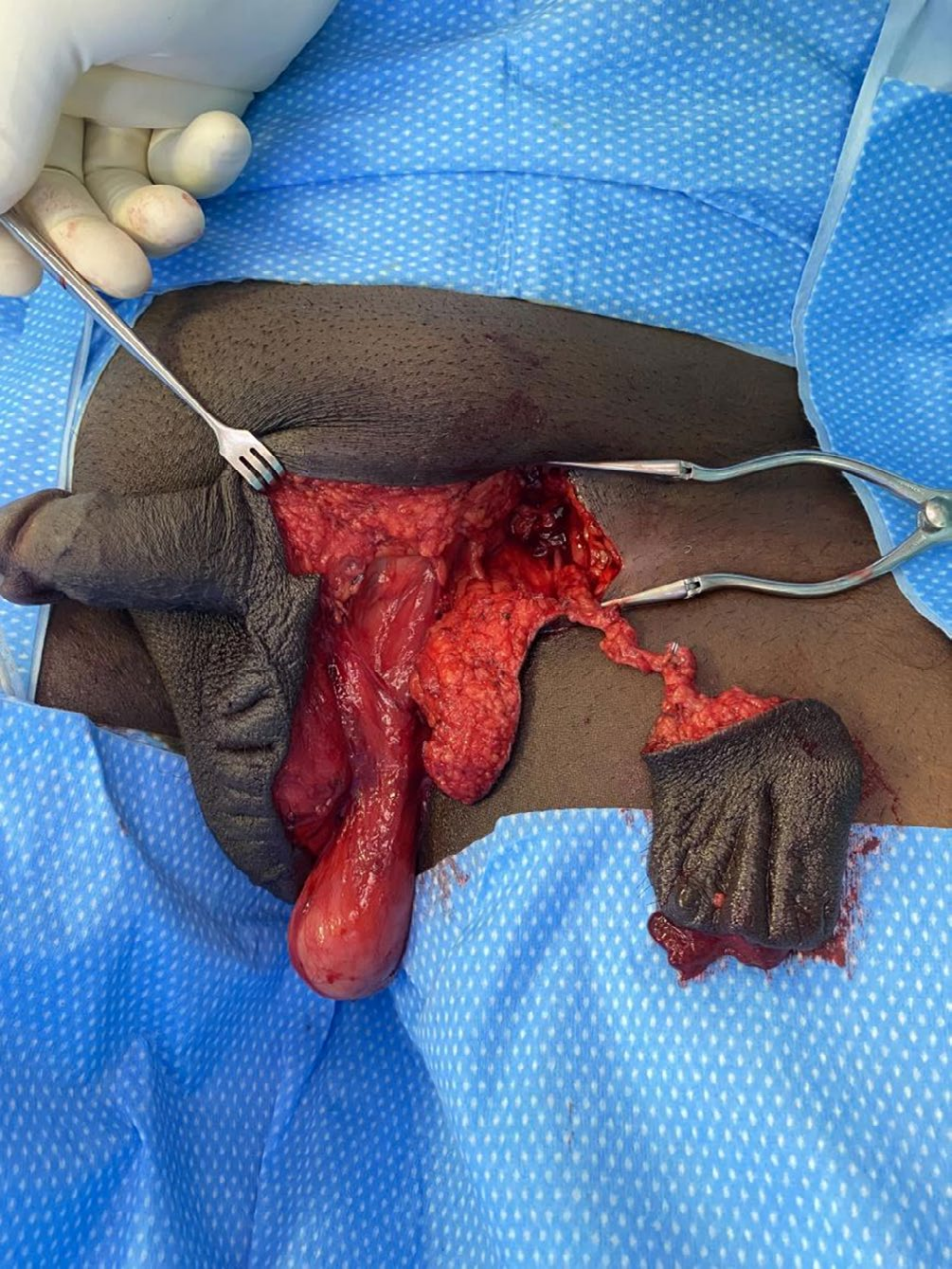
Flap harvest: Inguinoscrotal dissection and identification of key structures. Intraoperative view demonstrating elevation of the hemiscrotal fasciocutaneous flap: The testis and vas deferens temporarily delivered from the scrotal sac, the external pudendal pedicle dissected retrogradely toward its femoral origin, and the marked skin paddle designed for transfer. The flap is harvested in the plane of the external spermatic fascia.

**FIGURE 3:**
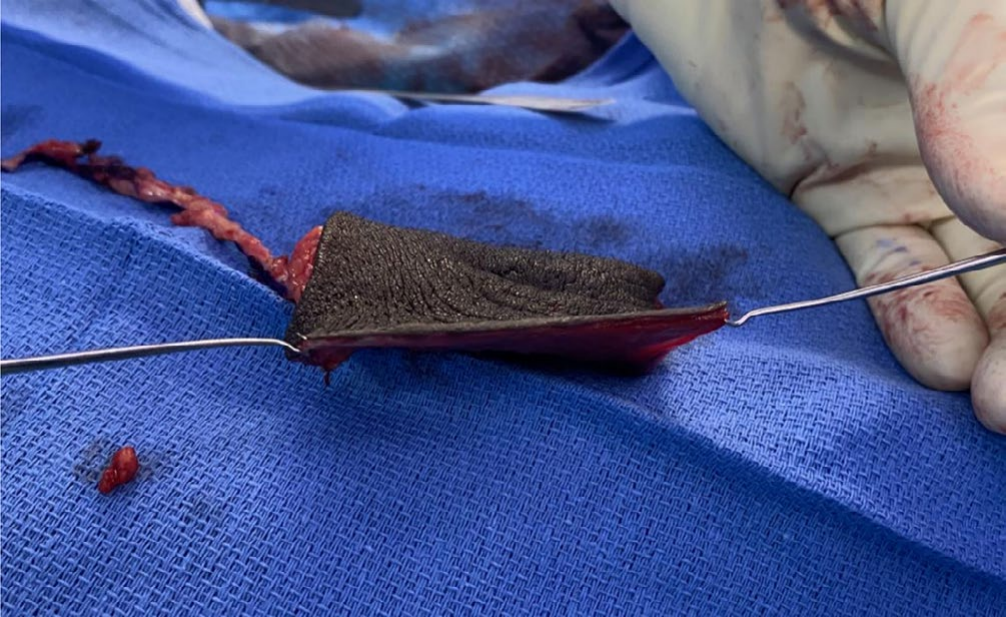
The harvested STEPA flap demonstrating its extremely thin and laminar architecture. The scrotal skin paddle, supplied by the external pudendal vessels, shows a uniform near‐millimetric thickness (approximately 2–3 mm), offering exceptional pliability and ideal characteristics for intraoral lining without the need for primary thinning.

A 6 × 6 cm fasciocutaneous paddle was designed across the left hemiscrotum and adjacent inguinal region. Through a curved incision along the inguinal crease, the suprapubic branch of the external pudendal artery was identified and dissected retrogradely to its femoral origin, providing a pedicle length of approximately 10–12 cm (Figure 1). The incision was extended toward the median raphe to access the anterior scrotal branches. The testis and spermatic cord were gently separated from the scrotal sac by atraumatic blunt dissection and temporarily displaced, allowing safe elevation of the hemiscrotal skin paddle (Figure 2). The flap was harvested in the plane of the external spermatic fascia, a naturally laminar layer yielding uniformly ultra‐thin tissue with minimal subcutaneous fat and excellent pliability (Figure 3).

**FIGURE 4:**
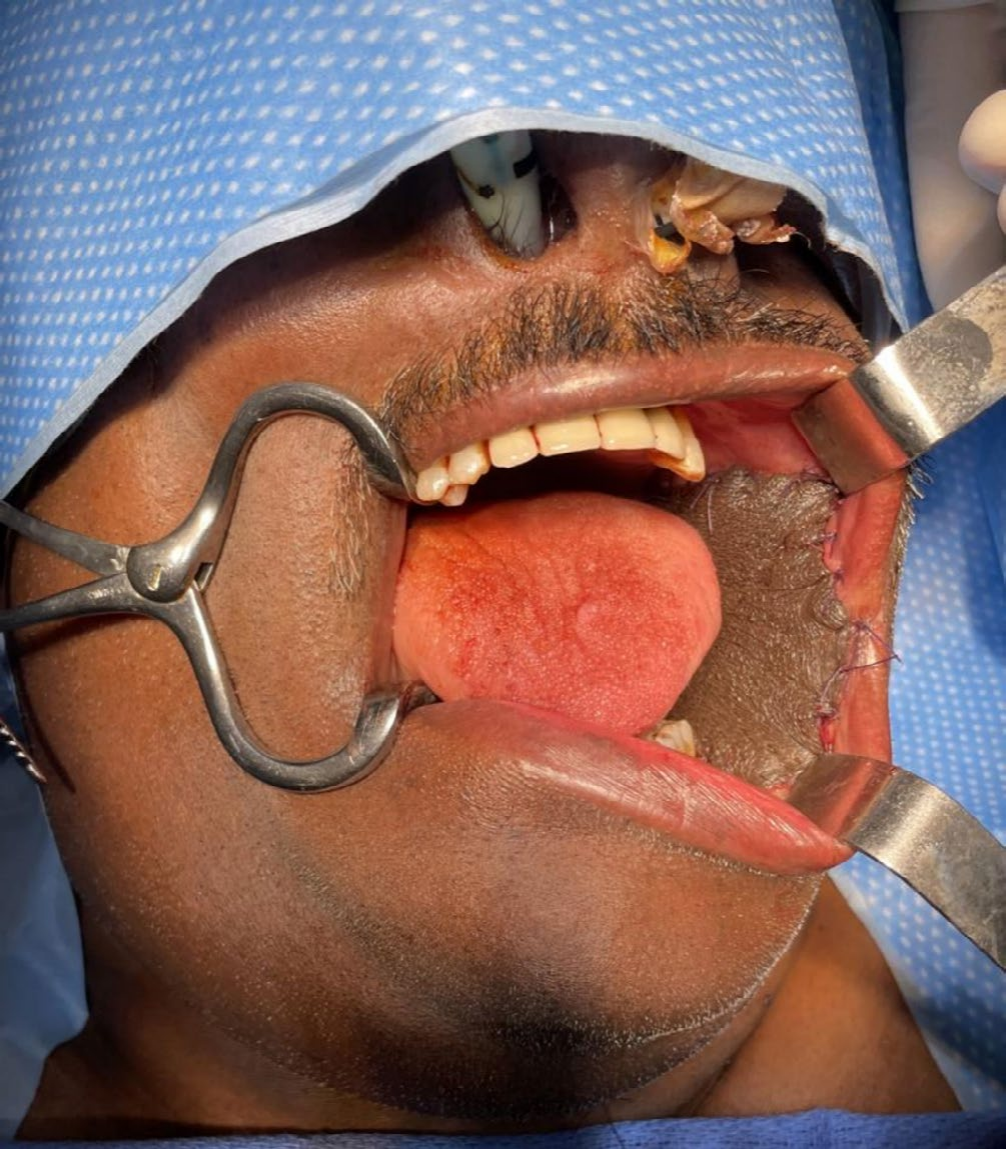
Intraoral inset of the STEPA flap. Immediate postoperative view showing the flap perfectly conforming to the left buccal mucosal defect. The thin, elastic hemiscrotal skin provides smooth lining, preserves the buccal sulcus and cheek mobility, and restores continuous mucosal coverage after wide oncologic excision.

After division of the pedicle and flap transfer, systematic orchidopexy was performed to prevent postoperative torsion, using fixation sutures between the tunica albuginea and the scrotal wall. The donor site was closed primarily without tension or distortion of the penoscrotal region. Microvascular anastomoses were performed end‐to‐end between the external pudendal artery and the facial artery, and between the external pudendal vein and the lingual branch of the common facial vein. Once revascularized, the flap was inset circumferentially to restore the buccal lining and cheek contour (Figure 4). The preserved parotid duct was marsupialized onto the superior posterior aspect of the flap.

The flap demonstrated immediate and stable perfusion. Enteral feeding was initiated on postoperative day 1, followed by progressive oral intake beginning around day 10. Early follow‐up showed complete integration of the flap, with preserved cheek mobility and satisfactory mouth opening. Donor‐site healing was uneventful, with no functional morbidity (Figure S1).

Final histopathology confirmed clear margins and absence of nodal metastasis (pT1N0), and surveillance without adjuvant treatment was recommended.

## 3 Discussion

This case illustrates the potential usefulness of the STEPA free flap in reconstruction of extensive buccal mucosa defects. The flap appears to offer a unique combination of thinness, elasticity, and pliability that is rarely matched by alternative donor sites. Compared to the radial forearm flap, the STEPA flap may help avoid graft‐related complications, conspicuous scarring, and functional limitations of the upper limb (Orlik et al. 2014), all particularly relevant in diabetic or manual‐labor patients.

To date, the clinical use of the STEPA free flap remains extremely limited, with only a single case reported in head and neck reconstruction (Dupret‐Bories et al. 2019) and no available clinical series. Data specifically regarding its use in oral cavity reconstruction remain particularly scarce. In this context, each additional case contributes meaningful clinical information regarding its feasibility and potential indications. Notably, this is, to our knowledge, the first report describing the use of the STEPA flap for buccal mucosal reconstruction, which represents a distinct anatomical and functional setting compared to previously reported oropharyngeal applications.

Beyond its favorable donor‐site profile, the main rationale for selecting the STEPA flap in this patient was the need for a reliably ultra‐thin and mobile lamina. Conventional “thin flaps” such as the ALT, TDAP, SCIP, and MSAP frequently show major variability in subcutaneous thickness, often exceeding several millimeters in high‐BMI patients and requiring systematic defatting to approximate mucosal thickness (Kim et al. 2018). In our obese patient, preoperative assessment confirmed that these flaps would be excessively thick and poorly suited for buccal lining, even with meticulous thinning, which would have increased the risk of fat necrosis and compromised perfusion. Anatomical studies of the external pudendal territory offer a striking contrast: Phoon et al. (2014) demonstrated a consistently near‐millimetric lamina (≈1.1 mm in over 80% of the flap), irrespective of body habitus, while Dupret‐Bories et al. (2019) and Benbassat et al. (2022) confirmed similarly thin and uniform architecture in clinical and cadaveric settings. These findings explain why the STEPA flap provided, in our patient, the only option capable of delivering a naturally ultra‐thin, homogeneous, and highly compliant tissue layer—properties essential for preserving cheek mobility, avoiding postoperative tightness, and restoring oral continence.

Another decisive advantage of the STEPA flap lies in the consistency and quality of its vascular pedicle. Phoon et al. (2014) described reliable arterial and venous diameters (mean 2.8 mm and 4.4 mm, respectively) and pedicle lengths of 10–12 cm, which greatly facilitate microvascular anastomosis—particularly in cervicofacial reconstruction where deep access angles are common (Phoon et al. 2014). Dupret‐Bories et al. (2019) reported similar calibers in clinical use, confirming the reproducibility of these measurements. More recently, Benbassat et al. (2022) demonstrated in 14 cadaveric dissections that the external pudendal artery is constant, with diameters ranging from 1.1 to 2.9 mm and pedicle lengths averaging 8.4 cm, thus validating its suitability for axial fasciocutaneous free flap design in both sexes. Together, these data highlight a pedicle comparable to those of ALT or RFFF flaps and particularly valuable in obese patients, for whom perforator anatomy is more variable (Shayan et al. 2008) and recipient‐site dissection more demanding.

As reported in prior literature, donor‐site morbidity remains minimal, with reliable primary closure, hidden scarring, and no negative impact on erectile or testicular function (Zhao et al. 2009; Swartz et al. 2007). Hair growth, the main drawback in intraoral use, can be managed by secondary laser depilation. In head and neck cancer reconstruction, however, this issue is often negligible, as most patients undergo adjuvant radiotherapy, which typically induces complete and permanent hair loss within the transferred flap. Pigmentation is of no functional consequence inside the oral cavity, and psychological concerns regarding scrotal harvest should be anticipated and discussed preoperatively.

Despite the superficial nature of the tumor (pT1), the indication for free flap reconstruction was driven by the wide extent of the mucosal defect and its functional implications. In this case, less invasive options such as skin grafts, buccal fat pad flaps, dermal substitutes, or locoregional mucosal flaps were considered but deemed suboptimal due to limited surface area, reduced elasticity, and the risk of postoperative contracture potentially impairing cheek mobility and oral continence (Habib and Medra 2016; Mardini et al. 2006). A free flap allowed reconstruction with a large, well‐vascularized and compliant lining, providing more predictable functional outcomes (Chien et al. 2005; Galviz Tabares et al. 2025). In addition, this procedure was performed in a high‐volume microsurgical center where free flap reconstruction is routinely carried out, supporting the use of a microsurgical approach in this context.

This case highlights the potential role of the STEPA free flap as an ultra‐thin reconstructive option in carefully selected patients. However, given the current paucity of clinical data, further cases, larger series, and comparative studies evaluating buccal reconstruction outcomes using local or regional techniques versus microvascular free flaps are needed to better define its indications, advantages, and long‐term outcomes.

## Funding

The authors have nothing to report.

## Ethics Statement

The authors have nothing to report.

## Conflicts of Interest

The authors declare no conflicts of interest.

## Supporting information


**Figure S1:** Clinical outcome at 3 months postoperatively. Left: Donor‐site appearance at the scrotal harvest site at 3 months, demonstrating minimal morbidity with well‐healed scars and no significant sequelae. Right: Intraoral view of the reconstructed buccal mucosa at 3 months, showing a thin, well‐integrated flap with preserved mouth opening. A minor refinement procedure is scheduled to correct a small excess of redundant tissue.

## Data Availability

The data that support the findings of this study are available from the corresponding author upon reasonable request.
