# Supplementary figures and images for: The Super Thin External Pudendal Artery Free Flap for Buccal Reconstruction: A Case Report
Source: Microsurgery. 2026 Jul 24;46(5):e70261. doi: 10.1002/micr.70261 (PMC13397553; doi:10.1002/micr.70261)

## Slide 1
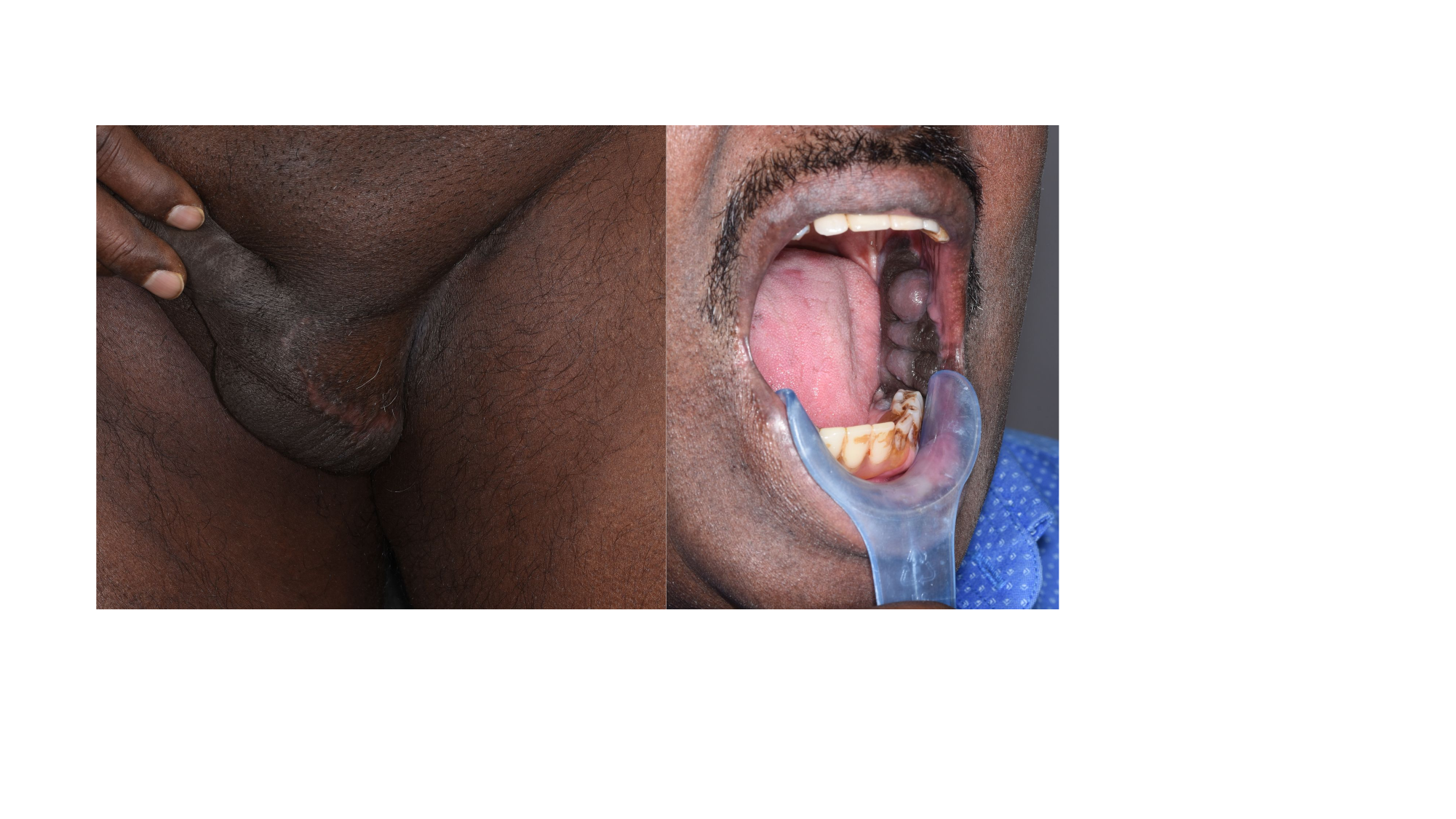

A
B
D

Supplement: Supplementary file 1 — Figure S1: Clinical outcome at 3 months postoperatively. Left: Donor‐site appearance at the scrotal harvest site at 3 months, demonstrating minimal morbidity with well‐healed scars and no significant sequelae. Right: Intraoral view of the reconstructed buccal mucosa at 3 months, showing a thin, well‐integrated flap with preserved mouth opening. A minor refinement procedure is scheduled to correct a small excess of redundant tissue. [file MICR-46-e70261-s001.pptx]
